# Selective redox signaling shapes plant–pathogen interactions

**DOI:** 10.1093/plphys/kiaa088

**Published:** 2021-01-23

**Authors:** Jade R Bleau, Steven H Spoel

**Affiliations:** Institute of Molecular Plant Sciences, School of Biological Sciences, University of Edinburgh, Edinburgh, EH9 3BF, UK

## Abstract

A review of recent progress in understanding the mechanisms whereby plants utilize selective and reversible redox signaling to establish immunity.

**Advances**

- Recent evidence highlights the importance of chloroplast-generated ROS production in response to pathogen infection.
- TRXs may confer selective redox signaling through chemical features and electrostatic complementarity
- New proteomic tools are beginning to uncover selective ROS/RNS signaling dynamics and the plant immune “redoxome.”
- Recent studies are advancing our understanding of how pathogens manipulate host redox systems.

## Introduction

Plants have evolved extensive signaling networks to regulate physiological responses to environmental stresses, both biotic and abiotic. Customary to many of these networks is the use of reactive oxygen and nitrogen species (ROS and RNS, respectively) as signaling tools. Signal-induced accumulation of ROS and RNS perturbs the homeostatic “redox state” of the cell (Spoel and Loake, 2011; Torres et al., 2006), which can result in detrimental protein oxidation and severe cellular damage. Therefore, it was widely assumed that ROS/RNS were toxic by-products of metabolism, whereas ROS/RNS scavenging antioxidant systems were seen as solely responsible for keeping undesirable ROS/RNS at low levels (Noctor, 2006). However, it is now established that ROS/RNS and antioxidant enzymes are vital signaling tools for intracellular and intercellular signaling and communication. Compared to other kingdoms, redox-related enzymes are particularly prevalent in plant genomes (Meyer et al., 2009; Birben et al., 2012; Geigenberger et al., 2017), suggesting plants have evolved numerous mechanisms to exploit ROS/RNS to their benefit. Indeed, ROS/RNS predominantly signal by oxidizing proteins. Metal chelating and aromatic amino acids are subject to irreversible oxidation, whereas methionine and relatively rare reactive cysteine (Cys) residues are reversibly oxidized in signaling proteins. ROS/RNS production and associated regulation by protein oxidation and particularly Cys oxidation are key aspects of plant immune responses. Extracellular perception of pathogen-associated molecular patterns (PAMPs) by pattern-recognition receptors as well as intracellular detection of pathogen effectors by nucleotide-binding leucine-rich repeat (NLR) immune receptors result in the production of ROS/RNS. But how these ROS/RNS selectively modify Cys residues to regulate specific signaling pathways remains largely unknown. Selective signaling is likely established by the diversity in ROS/RNS produced, their dynamic cellular location, ability to generate diverse oxidative Cys modifications, and the cellular distribution of different antioxidant systems. Here we discuss how redox signaling may be rendered selective and reversible in plant immune responses. Moreover, we explore how pathogens hijack host redox signaling and utilize their own antioxidant systems to promote virulence.

## Selective ROS/RNS production in response to pathogen detection 

**Figure 1:**
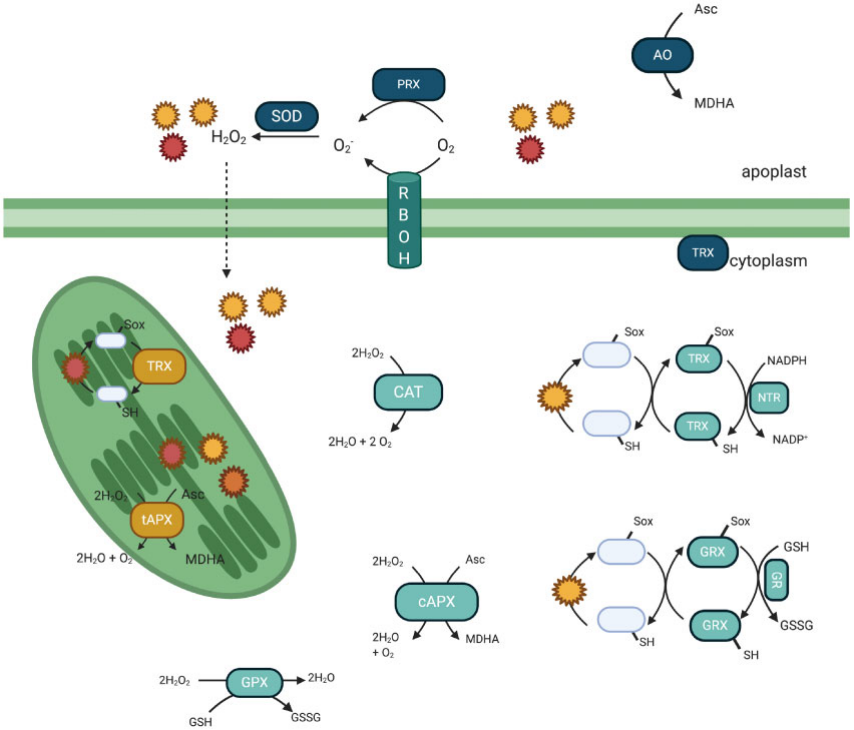
Localization and mechanisms of ROS/RNS production (signified by stars) and examples of known antioxidants and TRXs. ROS/RNS are produced in different subcellular locations, as are the antioxidant enzymes and oxidoreductases responsible for maintaining cellular redox homeostasis and reversal of oxidative protein modifications. Different cellular localization is indicative of selectivity and specificity in redox signaling. Created with BioRender.com. tAPX, thylakoid Asc PRX; cAPX, cytosolic Asc PRX; MDHA, monodehydroascorbate.

One of the earliest responses upon pathogen detection is the “oxidative burst,” resulting in the rapid generation and accumulation of ROS/RNS. Diverse reactive species may be produced, including singlet oxygen, superoxide ($O_{2}^{-}$), hydrogen peroxide (H_2_O_2_), hydroxyl radicals, nitric oxide (NO), and other NO derivatives. These molecules have distinct reactivities, different stabilities, and are produced by different cellular compartments, thereby generating the potential for selective signaling. The apoplast and the chloroplast are the main sites of ROS production upon pathogen detection (Figure 1). ROS/RNS can cause direct harm to the invading pathogen by triggering localized programmed cell death to prevent the spread of infection, callose deposition, and cross-linking of glycoproteins in cell walls (Torres et al., 2006; Daudi et al., 2012; O’Brien et al., 2012a), Alternatively, ROS/RNS act in cellular signaling or as long-distance signaling molecules via interaction with immune hormones such as salicylic acid (SA; Miller et al., 2009; Wang et al., 2014; Fichman and Mittler, 2020).

## Apoplastic ROS production

Pathogen-induced apoplastic ROS is largely produced by transmembrane localized Nicotinamide adenine dinucleotide phosphate (NADPH) oxidases, known as Respiratory Burst Oxidase Homologs (RBOHs). RBOHs transfer electrons from intracellular NADPH across the plasma membrane where they are coupled to molecular oxygen in the apoplast to produce $O_{2}^{-}$ (Figure 1; Suzuki et al., 2011). $O_{2}^{-}$ is short-lived and rapidly converted to H_2_O_2_, either spontaneously or by $O_{2}^{-}$ dismutase, and enters the cell through plasma membrane channels. The *Arabidopsis thaliana* genome contains 10 *RBOH* genes, with *RBOHD* and *RBOHF* being the main producers of ROS upon PAMP recognition and both are required for programmed cell death initiated after pathogen effector detection (Torres et al., 2002). RBOHs have been shown to have overlapping functions, but there is emerging evidence that they may also have more specific roles depending on the type of pathogen encountered. Indeed, D homolog and RBOHF show differential spatiotemporal expression levels and patterns in response to PAMPs, (hemi)biotrophic pathogens, and necrotrophic pathogens (Morales et al., 2016). A further clue that RBOH-mediated ROS production is finely regulated comes from its complex regulation by various post-translational modifications (PTMs), including phosphorylation. Both RBOHD and RBOHF are differentially phosphorylated at multiple residues by at least half a dozen different types of kinases that modulate their activity and stability (Kadota et al., 2015; Kimura et al., 2017, 2020; Han et al., 2019; Lee et al., 2020). These multiple post-translational control points suggest that ROS production and signaling can be selectively fine-tuned based on the type of pathogen encountered.

In addition to NADPH oxidases, apoplastic ROS can also be induced by peroxidases (PRXs). The Class III PRXs *PRX33* and *PRX34* are important in the activation of PAMP-triggered immunity, as knockdown mutants in Arabidopsis cell cultures show much reduced H_2_O_2_ levels in response to PAMP treatments and a decrease in expression of PAMP-induced defense-related proteins (O’Brien et al., 2012b). The absence of functional *PRX33* and *PRX34* resulted in increased susceptibility to the bacterial leaf pathogen *Pseudomonas syringae*, highlighting their role in early pathogen responses (Daudi et al., 2012). Moreover, the activity of ascorbate (Asc) oxidase (AO) reduces apoplastic amounts of the antioxidant Asc, thereby generating an environment in which apoplastic ROS can be sustained (Foyer et al., 2019). How the ROS-related activities of RBOH, PRX, and AO enzymes are balanced remains unknown, but their interplay likely generates specific ROS signatures depending on the stress encountered.

## Pathogen-induced ROS production by the chloroplast

Pathogen-induced apoplastic ROS production is well established and has been extensively reviewed in recent years (Kadota et al., 2015; Qi et al., 2017). However, the importance of organelles, particularly the chloroplast, in pathogen-induced ROS production and downstream immune signaling has only recently emerged. Chloroplasts are the source of both ROS and NO through, respectively, the electron transport chain and thylakoid-associated nitrite reductase or a NO-synthase-like enzyme (Figure 1; Lu and Yao, 2018). Indeed, PAMP perception leads to the second burst of ROS generated by the chloroplast that occurs after apoplastic ROS production (de Torres Zabala et al., 2015). Moreover, infection with a disabled *P. syringae* strain, deficient in delivering pathogen effectors into host cells, but not wild-type *P. syringae*, resulted in chloroplastic ROS production. As opposed to the wild-type, the disabled strain is largely noninfectious, suggesting that chloroplastic ROS may contribute to immunity. In accordance, it was also observed that inhibition of H_2_O_2_ production via photosystem II with 3-(3,4-dichlorophenyl)-1,1-dimethylurea, abolished ROS production and promoted virulence of this disabled pathogen (de Torres Zabala et al., 2015). In contrast to this hemibiotroph, chloroplastic ROS production has been shown to facilitate infection by the necrotrophic fungal pathogen *Botrytis cinerea.* By targeting a cyanobacterial antioxidant, flavodoxin, to the chloroplast to lower ROS accumulation, a decrease in tissue damage and hyphal growth was observed (Rossi et al., 2017).

Because chloroplasts have important roles in regulating localized cell death and nuclear gene expression (Zurbriggen et al., 2009; Straus et al., 2010), it is likely that the chloroplast-generated ROS signal is perceived throughout the cell. Using the genetically encoded biosensors Grx1-roGFP2 and roGFP2-Orp1 that, respectively, measure cellular glutathione redox potential and endogenous H_2_O_2_, it was indeed shown that methyl viologen-induced chloroplastic ROS generation led to rapid oxidation in the chloroplast and subsequently also in the cytosol and mitochondria (Ugalde et al., 2020). Thus, chloroplasts play a key role in orchestrating cellular responses to stress, including pathogen infection. This is perhaps best illustrated by the fact that upon pathogen infection, chloroplasts either congregate around the nucleus or send out dynamic extensions along microtubules called stromules that physically connect to the nucleus (Caplan et al., 2015; Kumar et al., 2018). Interestingly, exogenous application of H_2_O_2_ but not of NO or $O_{2}^{-}$, mimicked stromule formation induced by pathogen infection, indicating that this process is coordinated by specific ROS. Chloroplast rearrangements toward the nucleus may be a general response to infection, as Ding et al. (2019) observed this process after treatment of *Nicotiana benthamiana* with bacteria, viruses, bacterial elicitors, or exogenous H_2_O_2_. Interestingly, treatment with the NADPH-oxidase inhibitor diphenyleneiodonium, or the ROS scavengers dimethylthiourea and Tiron resulted in a drastic reduction in the percentage of nuclei surrounded by chloroplasts (Ding et al., 2019). These findings suggest that pathogen-induced H_2_O_2_ produced in the apoplast by NADPH oxidases signals to chloroplasts prior to the chloroplastic ROS burst.

## Pathogen-induced NO and NO derivatives

In animals, NO is one of the main RNS and is synthesized by dedicated NO synthetases (NOS). NO is formed by the NADPH-dependent oxidation of L-arginine to NO and citrulline. However, a bona fide NOS has not yet been identified in higher plants. Instead, in plants, NO is synthesized through alternative routes that have been reviewed in detail elsewhere (Astier et al., 2018a). One of the main sources of NO during the immune response is via nitrate reductases (NRs). The NR-deficient mutant *nia1nia2* in Arabidopsis provided insight into the importance of NO in response to pathogen infection. Lower levels of endogenous NO in *nia1nia2* mutants have been linked to regulation of oligogalacturonide-triggered immunity, elicitor-triggered immunity, and resistance to both the necrotrophic fungus *B. cinerea* and cucumber mosaic virus (Rasul et al., 2012; Jian et al., 2015). In addition, NO feedback regulates the nitrogen assimilation pathway, thereby controlling its own production (Frungillo et al., 2014). Pathogen infection leads not only to the accumulation of free NO but also of the NO-derivative *S*-nitrosoglutathione (GSNO). Both of these RNS play key roles in immunity as their respective overaccumulation in *NO Overexpressing 1* (*nox1*) and *GSNO Reductase 1* (*gsnor1*) mutants results in enhanced disease susceptibility (Feechan et al., 2005; Kneeshaw et al., 2014). While free NO⋅radicals are highly reactive and short-lived, GSNO is more stable and is thought to function as a cellular reservoir for NO bioactivity. The difference between these molecules in terms of molecular structure and mode of reactivity with Cys residues to form *S*-nitrosothiols (SNOs) suggests that they could have distinct roles in cell signaling. In accordance, upon pathogen infection, *gsnor1 nox1* double mutants displayed an increase in total cellular SNO upon pathogen perception and enhanced disease susceptibility compared to either of the single mutants (Yun et al., 2016). Moreover, overexpression of the GSNO scavenger enzyme *GSNOR1* rescued disease resistance in *gsnor1* but not in *nox1* mutants (Yun et al., 2016). These findings provide genetic evidence that RNS has distinct and selective signaling roles in plant immunity. The selective activities of ROS/RNS also cross-regulate each other to fine-tune redox signaling (Box 1; Lee et al., 2017).

Box 1 Cross-regulation of ROS/NO signaling

Increasing evidence has shown there is cross-talk between ROS and NO to tightly control redox signaling responses, including programmed cell death (Astier et al., 2018b). Following infection with an avirulent strain of *P. syringae*, treatment with the NO donors GSNO or CysNO substantial decreased NADPH oxidase activity, indicating that NO negatively regulates NADPH-mediated ROS production upon pathogen infection (Yun et al., 2011). Further analysis showed that RBOHD is *S*-nitrosylated at Cys890 and its mutation enhanced RBOHD activity in response to pathogen infection, further demonstrating that ROS/NO signaling is interlinked.

Additionally, early oxPTMs that function upstream of RBOHD-mediated ROS production may control downstream redox signaling mechanisms. RLKs regulate RBOHD-mediated ROS production (Kimura et al., 2017, 2020); however, it has also been suggested that RLKs themselves may be redox-regulated. The Cys-rich receptor kinase (CRK) subfamily of RLKs contains a high number of conserved Cys residues, suggesting that CRKs may be targets of redox regulation. In accordance, Lee et al. (2017) showed that overexpression of CRK36 enhanced the cell death response and increased stomatal immunity and resistance to hemibiotrophic as well as necrotrophic pathogens, but overexpression of a Cys mutant abolished responses. Furthermore, the authors showed that CRK36 interacted with the receptor-like cytoplasmic kinase BIK1, which is responsible for activating RBOHD upon PAMP-perception, suggesting that early redox modifications of CRKs could control downstream ROS production.

H_2_O_2_ and NO have also been detected in distal tissues following infection, and mutants compromised in ROS or NO biosynthesis were found to be compromised in systemic acquired resistance (SAR), indicating ROS/NO signaling is also involved in long-distance immune signaling (Wang et al., 2014). Interestingly, NO levels in the ROS biosynthesis mutant *rbohF* were undetectable after pathogen infection, likewise the NO biosynthesis mutant *noa1 nia2* displayed reduced levels of ROS after pathogen infection, suggesting that ROS/NO-mediated SAR is interdependent and may be regulated by a ROS/NO feedback loop (Wang et al., 2014).

## Oxidative PTMs orchestrate immunity

Pathogen-induced ROS/RNS alters the redox state of the cell by changing the oxidative status of small redox couples, including oxidized versus reduced glutathione (Spoel and Loake, 2011). Such redox changes can be sensed directly by signaling proteins of the immune system. For example, in unchallenged cells, the immune transcription co-activator nonexpressor of pathogenesis-related genes 1 (NPR1) resides in the cytoplasm as a disulfide-linked oligomer. Upon infection, an increasingly reduced cellular environment results in release of NPR1 monomers either by spontaneous reduction of disulfides (S–S) or their cleavage by specific oxidoreductases (Mou et al., 2003; Tada et al., 2008). NPR1 monomers then translocates to the nucleus where they orchestrate transcriptional reprogramming to prioritize immunity over other cellular processes (Kinkema et al., 2000; Wang et al., 2006). Signaling proteins like NPR1 may therefore be thought of as “redox sensors” that sense and respond to the cellular redox environment.

ROS/RNS can also directly oxidize reactive Cys residues of signaling proteins (Waszczak et al., 2015). This results in oxidative PTMs (oxPTMs) of diverse nature, including protein *S*-nitrosylation (–SNO), S–S, *S*-sulfhydration, *S*-sulfenylation (–SOH), *S*-sulfination, and *S*-sulfonation. Except for the latter, all other oxPTMs are reversible either through small molecule—or enzymatic reduction. Reversible oxPTMs can also occur on methionine, another sulfur-containing amino acid. Unlike Cys oxidation, which is pH-dependent and requires its thiols to be ionized for oxidation to occur, methionine oxidation to methionine sulfoxide occurs over a broad pH range (Levine et al., 2000; Peskin and Winterbourn, 2001; Kim et al., 2014). Thus, oxPTMs ensure that ROS/RNS signatures are translated into dynamic molecular processes. Recent work demonstrated that oxPTMs underpin perception of apoplastic H_2_O_2_ at the cell surface. A forward genetic screen based on imaging H_2_O_2_-induced calcium fluxes identified the plasma membrane-localized leucine-rich repeat receptor kinase H_2_O_2_-induced Ca^2+^ increases (HPCA1; Wu et al., 2020). HPCA1 contains extracellular Cys residues that are oxidized upon exposure to H_2_O_2_, leading to its activation by autophosphorylation and downstream induction of calcium channels and stomatal closure. While currently known receptor-like kinases (RLKs) involved in pathogen perception did not respond to H_2_O_2_ in a similar manner (Wu et al., 2020), this mechanism of H_2_O_2_ perception appears to be conserved and could mediate H_2_O_2_ responses of yet unknown pathogen receptors.

Upon pathogen infection, intracellular ROS/RNS target numerous signaling proteins involved in a wide variety of cellular processes (Waszczak et al., 2015; Camejo et al., 2019). Thus, discovering ways to detect oxPTMs on a global proteomic scale is an important step forward toward uncovering how ROS/RNS regulates immune signaling proteins. Several key biochemical and proteomic methodologies have emerged over the past several years that have provided a global view of how pathogen-induced ROS/RNS may control cell signaling. Waszczak et al. (2014) developed a probe based on the C-terminal Cys-rich domain of the yeast AP-1-like (YAP1C) transcription factor and were able to uncover the H_2_O_2_-dependent sulfenome (i.e. proteins that have a least one Cys that has been modified by sulfenic acid). The authors detected a range of proteins in the first 10 min of H_2_O_2_-induced oxidative stress, including proteins involved in signal perception and transduction such as mitogen-activated protein kinases (MAPKs), as well as redox-related proteins such as thioredoxin (TRX)-dependent PRX1 and dehydroascorbate reductase 2 that is involved in the H_2_O_2_ scavenging pathway. This study provided one of the first deep insights into the array of redox-mediated signaling pathways and networks that can be uncovered with redox-specific proteomic tools. Further development of the YAP1C probe now allows it to be expressed *in vivo* in specific cellular locations, including the chloroplast (De Smet et al., 2019). Such organellar proteomic approaches will be highly useful for understanding how the pathogen-induced apoplastic H_2_O_2_ burst is perceived intracellularly by proteins of the immune system. A drawback of the YAP1C probe is that it only identifies peptides that interact with the probe but not the identities of *S*-sulfenylated amino acids. A recent technological advancement now overcomes this limitation by using an anti-YAP1C-derived peptide antibody to purify YAP1C cross-linked target peptides, identifying *S*-sulfenylated Cys residues in ˃1,000 proteins (Wei et al., 2020).

An emerging method for uncovering global oxPTMs, or the “redoxome,” is the use of chemical proteomics using Cys-reactive chemical probes, including activity-based protein profiling (ABPP), to detect changes in the activities of proteins on a proteome-wide scale. ABPP and other Cys-reactive proteomic methods commonly use probes that label the active site of enzymes, but are also suitable for labeling highly reactive Cys residues (Morimoto and van der Hoorn, 2016; Foyer et al., 2019). ABPP labeling is often irreversible and enables modified proteins or peptides to be purified and identified by mass spectrometry (Morimoto and van der Hoorn, 2016). Although successfully applied in plants, one of the limitations to ABPP is the lack of specificity it provides in terms of the type of oxPTM that is detected. However, more selective probes for specific oxPTMs are now emerging. Using an SNO-specific biotin probing method, Hu et al. (2015) identified over 900 endogenously *S*-nitrosylated proteins in the *gsnor1-3* mutant that overaccumulates SNOs. Moreover, using a benzothiazinedioxide-based probe in Arabidopsis cell cultures to identify *S*-sulfenylated proteins after H_2_O_2_ treatment, identified 1,537 –SOH sites, including specific sites of immune-associated MAPK4, which when mutated, inhibit its phosphorylative activity (Huang et al., 2019). Further development of probes has now enabled the identification of specific modified Cys residues. Additional probes have been developed to enable the identification of free thiols, and other oxPTMs, including SNOs, sulfinic acids, and sulfhydrates, which can be applied to study plant immune signaling (Seneviratne et al., 2016; Akter et al., 2018; Zivanovic et al., 2019). However, specific probes have yet to be described for sulfonic acids, while detection methods for S–S bonds and glutathionylates are still primarily based on monocysteinic oxidoreductase substrate traps (Motohashi et al., 2001; Pérez-Pérez et al., 2017).

Recently, a label-free method uncovered Cys reactivity across the proteome during the immune response. McConnell et al. (2019) studied the effects of pathogen effector-triggered immunity on Cys oxidation in wild-type plants and the *top1top2* double mutant, which is deficient in thimet oligopeptidases (TOP1 and TOP2) that are involved in regulation of the oxidative burst and programmed cell death. This study demonstrated that TOP1/2 reinforced cellular oxidation dynamics upon immune activation and identified a wide repertoire of new oxPTMs involved in among others cell wall reinforcement and programmed cell death.

## Selective and reversible oxidative signaling

Considering the wide variety of oxPTMs and their ability to change protein function or activity, how are these modifications tightly controlled to enable cell signaling and to protect from protein hyperoxidation? First, the chemistry of each ROS/RNS and the accessibility of their Cys targets play an important role. Protein conformation will selectively exclude access for some ROS/RNS but not others, while residues surrounding the oxPTM can stabilize or destabilize the oxidative group, thereby determining oxPTM turnover times. Second, the spatial and temporal production of ROS can selectively expose Cys residues to ROS/RNS. As described above for the plant immune system, the activities of enzymes such as NADPH oxidases are indeed tightly controlled by numerous PTMs to ensure temporal production. Third, cellular scavenging or antioxidant systems also determine the spatiotemporal effects of ROS/RNS on Cys residues. A recent study demonstrated that the glutathione antioxidant system determines the cytosolic effects of the apoplastic ROS burst induced by perception of pathogen patterns. In the absence of cytosolic glutathione reductase 1, cytosolic H_2_O_2_ levels increased earlier and were of higher amplitude, indicating that in wild-type plants (reduced) glutathione effectively scavenged H_2_O_2_ (Nietzel et al., 2019), thereby likely defining the spatiotemporal dynamics of ROS-mediated oxPTMs. In addition, ROS scavenging enzymes such as catalase (CAT) and PRX enzymes, including Asc PRXs (APX), peroxiredoxins, and glutathione PRXs (GPX), have been shown to play roles in the immune response (Figure 1; Choi et al., 2007; Daudi et al., 2012; Survila et al., 2016; Yuan et al., 2017). Silencing of the thylakoid-localized H_2_O_2_ scavenging enzyme tAPX resulted in the activation of defense-related genes, including *ICS2,* which is responsible for SA biosynthesis and downstream expression of *pathogenesis-related* genes (Maruta et al., 2012). Additionally, CAT2 plays a role in hormonal immune signaling. Yuan et al. (2017) reported that CAT2 may function as an SA receptor, whereby SA inhibited its H_2_O_2_ scavenging activity. Decreased activity of SA-bound CAT2 and associated increases in H_2_O_2_ levels inhibited auxin and jasmonic acid biosynthesis enzymes, thereby alleviating the negative effects of these hormones on SA-mediated resistance to biotrophic pathogens. Thus, ROS scavenging enzymes may represent one of the missing links between hormone and ROS signaling, and have the potential to selectively shape the oxidative proteome. The potential of ROS/RNS scavenging enzymes to shape the oxidative proteome is clearly illustrated by the GSNO scavenger GSNOR1. In the absence of functional GSNOR1, cellular levels of GSNO and consequently, protein –SNO markedly increase, especially upon pathogen infection (Feechan et al., 2005; Yun et al., 2011).

While these scavenging enzymes are important in directly regulating ROS/RNS and oxidative stress, particularly during pathogen infection, there is another group of enzymes, known as oxidoreductases or redoxins, which are responsible for direct reversal of ROS/RNS-induced oxPTMs. These enzymes belong to the superfamily of TRXs that are emerging as central players in selective redox signaling.

## TRX-mediated immune responses

TRXs are a superfamily of oxidoreductase enzymes. They are made up of conventional TRXs, glutaredoxins (GRXs), nucleoredoxins (NRXs), and protein disulfide isomerases (PDIs; Meyer et al., 2008; Marchal et al., 2014). Conventional TRXs have a conserved active site sequence WC(G/P)PC (Meyer et al., 2008). One of the key functions of conventional TRX enzymes is to reduce S–S bonds in target proteins. S–S bridges are reduced by a dithiol mechanism; the first active-site Cys covalently binds to the Cys of the target protein to form a mixed S–S, before the second active-site Cys resolves the mixed S–S, thereby releasing the reduced target protein while rendering the TRX active-site Cys residues oxidized (i.e. S–S bridge). Over the last several years, it has become clear that TRXs not only reduce S–S bonds but also other oxidized Cys forms, including SNOs and sulfenic acids, via either heterolytic or homolytic cleavage reactions (Benhar et al., 2008; Tarrago et al., 2010; Kneeshaw et al., 2014). TRX activity is recycled by NADPH-dependent TRX reductases (NTRs) or ferredoxin-dependent TRX reductases (FTR) that reduce oxidized TRXs by donating electrons to NAD(P)H (Figure 1).

The TRX family has been shown to play diverse mechanistic roles in plant immune responses. Their mechanisms of action range from exhibiting direct anti-microbial activity, to reversing oxPTMs to either enable signaling or to protect critical proteins from the damaging effects of oxidation. With regard to the former, OsTRXm and the TRX-like protein OsTDX from *Oryza sativa* were found to display anti-microbial activity (Park et al., 2019a, 2019b). OsTRXm was identified as a novel antifungal protein, inhibiting the growth of several fungi, including the maize pathogen *Fusarium moniliforme* (Park et al., 2019b). Although TRX*h*5 is not secreted and thus not considered to be a natural antifungal protein, recombinant TRX*h*5 from Arabidopsis was found to display antifungal properties. Interestingly, when the yeast *Candida albicans* was treated with AtTRX*h*5, it exhibited an increase in ROS generation, suggesting that TRX*h*5 inhibited fungal growth by eliciting ROS production (Park et al., 2017). The identification of antifungal properties associated with TRX*h*5 suggests that there is scope for using its redox properties as a biotechnological crop improvement strategy.

TRX*h*5 and closely related TRX*h*3 have also emerged as major regulators of plant immune signaling (Laloi et al., 2004; Tada et al., 2008; Kneeshaw et al., 2014). Specifically, these TRX regulate the conformational state and thus activity of the NPR1 immune transcriptional activator. As described above, in resting cells NPR1 exists in an oligomeric form in the cytoplasm bound together by S–S bridges. Upon pathogen-induced SA accumulation, TRX*h*3/*h*5 facilitates NPR1 S–S reduction and associated monomerization. Moreover, TRX*h*5 directly denitrosylates NPR1, which prevents the formation of further S–S bridges (Tada et al., 2008; Kneeshaw et al., 2014). Consequently, TRX*h*5 plays a critical role in NPR1-dependent transcriptional reprogramming.

In addition to regulating protein signaling, TRXs have been shown to exhibit protective roles in immunity. This was shown recently for the immune inducible TRX family member NRX1 that can be found in both the cytoplasm and nucleus (Marchal et al., 2014). The *nrx1* knock-out mutant displays autoimmune traits and has increased resistance to *P. syringae* compared to wild-type Arabidopsis (Kneeshaw et al., 2017). Using a substrate-trapping approach, NRX1 was found to target H_2_O_2_ scavenging enzymes, including all three Arabidopsis CATs and APX1, all of which play roles in the immune response (Kneeshaw et al., 2017). The authors showed that CAT2 oxidation in response to bacterial infection was enhanced in the *nrx1-1* knockout mutant, which correlated with a decrease in CAT activity. Moreover, recombinant NRX1 could rescue CAT activity of *nrx1-1* cell extracts. These findings demonstrate that NRX1 prevents inhibitory oxidation of CATs, thereby guarding them from the ROS-rich environments they function in. Taken together with the fact that TRX family members play essential roles in recycling the activity of peroxiredoxins that also scavenge H_2_O_2_, it appears justified to conclude that protection of ROS scavengers and probably other types of proteins is a general function of TRX family members.

So how are the activities of TRX enzymes regulated? Little remains known about the regulation of TRX. In addition to regulation of TRX gene expression, TRX enzymes may also be redox-regulated. Plant and human TRX*f* and TRX-1, respectively, are regulated by glutathionylation. Glutathionylation decreases the ability of plant TRX*f* to be reduced by ferredoxin-TRX reductase, while human TRX-1 was inactivated. These findings suggest that cellular redox status can control the activity of some TRX enzymes, but this may not be a widespread mechanism utilized by all TRX enzymes (Casagrande et al., 2002; Michelet et al., 2005).

## TRX substrate specificity in the immune response

In contrast to other organisms, plant genomes encode numerous TRXs. The high number of TRXs in plants and the range of locations in the cell suggests that they have specific cellular functions and substrates (Fernandes and Holmgren, 2004; Geigenberger et al., 2017). TRXs are characterized by their highly conserved active site sequence and by relatively large groups of members residing in the same subcellular location. So do TRX act selectively and if so, what determines their substrate specificity?

As there is redundancy between TRXs as well as between the GRX and TRX systems (Reichheld et al., 2009), knockout mutants often do not show the full extent of TRX function, thereby preventing identification of specific substrate repertoires. One method of studying TRX substrate selectivity is by overexpressing them in redox-perturbed mutants. The effectiveness of this approach was demonstrated by overexpression of immune-induced TRX*h*5 in two different protein –SNO accumulating backgrounds: *gsnor1* and *nox1* mutants that, respectively, overaccumulate GSNO and free NO (Kneeshaw et al., 2014). In response to *P. syringae* infection, TRX*h*5 expression selectively rescued immune deficiencies in *nox1* but not *gsnor1*. This was due to selective denitrosylation of protein–SNO, as TRX*h*5 localized to similar subcellular locations but decreased protein–SNO concentrations only in *nox1* mutants. These findings indicate that TRX*h*5 can distinguish between protein–SNO derived from free NO as opposed to GSNO.

**Figure 2:**
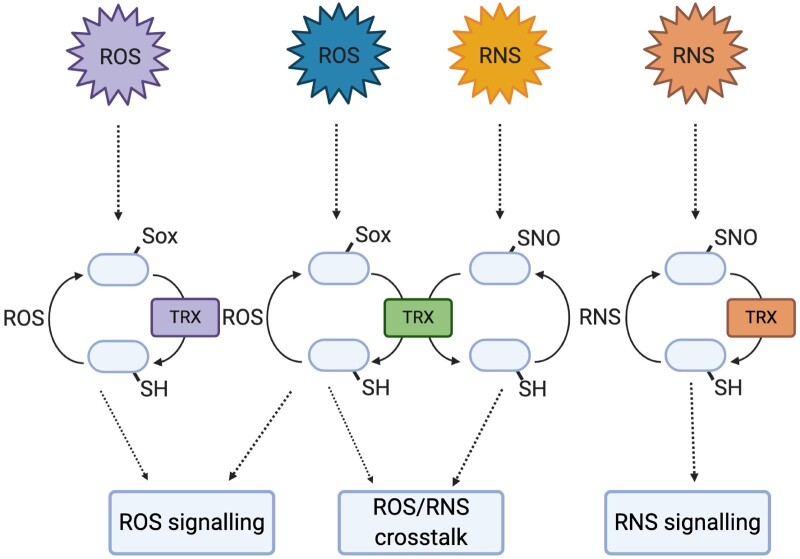
Selective redox signaling in the immune response. Upon pathogen perception, different forms of ROS/RNS are generated, resulting in oxidative modifications of the reactive free thiols of “redox sensitive” proteins. Modified proteins may be involved in specific ROS/RNS-mediated signaling pathways, or overlap with different pathways. These modifications can be reversed by specific TRXs, adding an additional layer of selective regulation to ROS/RNS-mediated signaling. TRXs may also display selective tendencies for targets involved in specific pathways or display more general reductive activities. Created with BioRender.com.

How exactly such substrate specificity is established remains poorly understood, but several recent studies on molecular characteristics of both TRXs and their substrates are now beginning to shed light on this. It has been shown that stereochemistry and structure of NO donors is a major determinant in selective *S*-nitrosylation, due to Cys accessibility in the 3D structure of target proteins (Foster et al., 2009). It is therefore reasonable to assume that the structural conformation of substrates will play an important role in their selection by specific TRXs. In accordance, domain swapping between TRXs enhanced their antifungal activity, showing that structural differences between TRXs can lead to functional changes (Kim et al., 2019). Moreover, it was shown that due to conformational restrictions imposed by S–S bonds on the substrate, TRXs recognize oxidized Cys with greater efficiency than its reduced counterpart (Palde and Carroll, 2015). If this is also true for oxidation states other than S–S bonds remain to be explored. Another possibility is that specificity is determined through electrostatic complementarity between TRX and its substrate. Although the TRX active site is evolutionarily highly conserved, the electrostatic potential of the surrounding solution-exposed surfaces shows variation. Gellert et al. (2019) assessed the primary and tertiary structure of human TRXs and GRXs, and found that primary and tertiary structure did not contribute to substrate specificity. Instead, they show that TRXs and GRXs could be grouped based on the positive or negative electrostatic potentials of their water-accessible surfaces, suggesting this may determine substrate specificity. Indeed, the *Chlamydomonas reinhardtii* TRX CrTRXf1 was shown to have an electrostatic positive crown that corresponds to the electronegative surfaces surrounding the Cys residues of its substrates FBPase and SBPase (Lemaire et al., 2018), adding weight to this hypothesis. Hence, a combination of localization, structural accessibility or flexibility, and electrostatic complementarity will likely determine specific TRX substrate repertoires (Figure 2).

## Pathogens manipulate and hijack redox-dependent immune responses

Redox-mediated immune responses to pathogen infection effectively prevent pathogen spread. However, the arms race between pathogen and host can lead to pathogens overcoming host defense responses. Recent evidence indicates that pathogens not only utilize their own redox systems to cope with host immune responses (Box 2; Dubreuil et al., 2011; Viefhues et al., 2014; Habash et al., 2017; Jain et al., 2018; Zhang et al., 2019; Gross et al., 2020), but they also manipulate and exploit cellular redox responses of the host.

Box 2 Pathogen TRX systems aid virulence

Emerging evidence shows pathogens utilize TRX systems of their own, which can enhance their ability to infect host plants. Both the nematode *M. incognita* and the bacterium *Candidatus Liberbacter asiaticus* utilize TRX-dependent PRXs to enable infection (Dubreuil et al., 2011; Jain et al., 2018). Additionally, putative PDIs have also been identified in plant–parasitic nematodes, including *Heterodera schachtii* and *Globodera pallida* (Habash et al., 2017; Gross et al., 2020). Recently, the *G. pallida* effector GpPDI1 was characterized as a functional thioredoxin. When transiently expressed in *N. benthamiana* and tomato it was found to induce cell death, however, it was also found that its enzymatic activity was not required for the host elicitation of cell death, which suggests its TRX-like activity may not be required for host recognition (Gross et al., 2020).

Although necrotrophic pathogens can benefit from ROS/RNS induced cell death in the host, they still need to find a way to function in highly oxidative environments, suggesting they may require their own TRX systems. This has been demonstrated in the necrotrophic pathogen *Scelortinia sclerotiorum*, which required the TRX reductase SsTrr1 for increased oxidative stress tolerance and virulence (Zhang et al., 2019). Exogenous H_2_O_2_ treatment of *SsTrr1*-silenced strains resulted in inhibition of hyphal growth and decreased virulence in Arabidopsis and *N. benthamiana*, suggesting that it is crucial for surviving the ROS burst upon infection (Viefhues et al., 2014; Zhang et al., 2019). Similar results were obtained for *B. cinerea* in that knockout of *BcTRX1* and *BcTRX2* weakened virulence, while increasing sensitivity to oxidative stress. Together these findings indicate that pathogen TRX systems are highly important for aiding pathogen virulence.

The production of ROS and RNS are important weapons of eukaryotic immune systems, either directly killing pathogens by causing oxidative stress or by activating host immune signaling. Consequently, many pathogens have evolved effector proteins that suppress the immune-induced oxidative burst. For example, the *P. syringae* effector HopM1, which targets vesicle trafficking, was shown to also inhibit the immune-induced ROS burst via a proteasome-dependent pathway (Lozano‐Durán et al., 2014). Indeed, HopM1 interacts with proteasomes and inhibits their proteolytic activity (Üstün et al., 2016). Moreover, several *P. syrinagae* effectors suppress RLKs that activate NADPH oxidases and thus circumvent not only pathogen perception, but also the damaging effects of the ROS burst (Xin et al., 2018). Some pathogen effectors directly alter the activity of plant redox enzymes. For example, Pep1 is a conserved fungal effector of smut-causing fungi on cereals and was found to directly inhibit the activity of apoplastic plant PRXs, thereby boosting pathogen virulence by suppressing the apoplastic ROS burst (Hemetsberger et al., 2015).

In addition, ROS scavenging enzymes, including CATs and glutathione S*-*transferases, have been identified during infection as pathogen- and parasite-secreted proteins (Zhang et al., 2004; Dubreuil et al., 2007; Blackman and Hardham, 2008; Tanabe et al., 2010; Guo et al., 2012). These enzymes may function as effectors that enable pathogen survival in hostile, oxidative host environments. Nonenzymatic antioxidants, such as the carbohydrate mannitol, are also secreted by some pathogens and may also combat the antimicrobial effects caused by high levels of ROS/RNS in host cells (Jennings et al., 2002; Voegele et al., 2005; Williamson et al., 2013)*.* Thus, pathogen manipulation of host redox responses appears to be a widespread mechanism for promoting pathogen virulence.

Recent studies have shown that pathogen effectors also suppress ROS production by targeting the chloroplast, leading to associated reductions in programmed cell death, photosynthesis, electron transport, and PSII activity (Rodríguez-Herva et al., 2012; de Torres Zabala et al., 2015; Xu et al., 2019). This was further demonstrated in the case of the *Phytophthora infestans* effector AVRvnt1 and its NLR immune receptor Rpi-vnt1.1, which activates immunity in a light-dependent manner (Gao et al., 2020). The authors reported that in Solanaceous plants, the AVRvnt1 effector interacted with the nuclear-encoded chloroplast protein Glycerate Kinase (GLYK), which was required for Rpi-vnt1.1-mediated immunity. GLYK was found to be a positive regulator of plant immunity, but exposure to light was required to produce full-length GLYK, including its chloroplast transit peptide. AVRvnt1 prevented the accumulation of GLYK and may intercept GLYK trafficking to the chloroplast. As GLYK is involved in energy production and photorespiration, the perturbation of canonical GLYK activity by AVRvnt1 likely alters redox-mediated immune responses in the chloroplast by interfering with energy production.

Pathogens have also evolved ways to hijack redox-mediated immune responses through manipulation of host TRX systems. The RipAY effector from the plant pathogen *Ralstonia solanacearum* functions as a γ-glutamyl cyclotransferase, responsible for degradation of the cellular antioxidant glutathione that is required for immunity (Dubreuil-Maurizi et al., 2011). Interestingly, RipAY was found to interact with host TRX*h*3 and TRX*h*5, which were required for its activation and for the resulting degradation of glutathione and inhibition of immune responses (Fujiwara et al., 2016; Mukaihara et al., 2016; Sang et al., 2018). Both active site Cys residues of TRX*h*5 were required for interaction with RipAY and for its activation, indicating that *R. solanacearum* exploits the redox-sensing or oxidoreductase activity of host TRXs (Fujiwara et al., 2016, 2020).

Nematode effectors also target ROS-scavenging TRX systems of their hosts. The effector MjTTL5 secreted by the nematode *Meloidogyne javanica* was shown to interact with the Arabidopsis ferredoxin-TRX reductase catalytic subunit (AtFTRc). Mutational analyses showed that MjTTL5 and host AtFTRc both enhanced nematode susceptibility (Lin et al., 2016). Moreover, MjTTL5 suppressed the immune-induced ROS burst, suggesting that it exploits host AtFTRc to promote ROS scavenging and decrease oxidative stress. Similarly, the root-knot nematode *Meloidogyne incognita* was reported to overcome resistance by potentially exploiting PRXs in tomato plants. Whereas resistant tomato plants carry the *Mi-1* resistance gene, susceptible varieties of tomato show higher expression of genes involved in ROS scavenging, including glutathione S-transferase (*SlGST*) and PRX (*SlPOD*). Silencing of *SlPOD* in *Mi-1* carrying resistant varieties, resulted in an enhanced ROS burst upon infection in conjunction with a decrease in female nematode formation and egg mass, suggesting that *M. incognita* and probably other root-knot nematodes may confer susceptibility by exploiting ROS scavenging activities of host PRXs (Guan et al., 2017).

As an alternative to suppressing ROS accumulation, it has been shown that effectors of necrotrophic pathogens specifically target the plant TRX system to induce cell death responses. Victorin, a fungal effector of *Cochliobolus victoriae*, the pathogen responsible for Victoria blight in oat, specifically targets Arabidopsis TRX*h*5. Victorin binds to TRX*h*5 via the first Cys of its active site (Cys39), resulting in inhibition of TRX*h*5 activity (Sweat and Wolpert, 2007). This is recognized by the NB-LRR, Locus Orchestrating Victorin effects 1 (LOV1), which guards TRX*h*5, and in turn activates programmed cell death (Lorang et al., 2007, 2012; Sweat and Wolpert, 2007). As necrotrophs kill host cells before feeding, TRX*h*5-victorin-induced cell death promotes disease susceptibility to *C. victoriae*. Consequently, victorin inhibits TRX*h*5 activity and can cause susceptibility to biotrophic pathogens, providing a clue as to why LOV1 guards TRX*h*5 and demonstrating that *C. victoriae* hijacks a redox-dependent immune response to biotrophs to promote its own virulence. Moreover, these findings suggest that TRX enzymes may be more widely targeted by pathogen effectors.

## Conclusions

Redox-based signaling is a vital part of plant immune responses and new redox mechanisms are continuously being discovered. Recent studies have provided new insights and hypotheses of how “redox sensors” may be regulated during the plant immune response by the type of ROS/RNS they are exposed to, and by specific ROS/RNS scavenging enzymes and TRXs. However, how these vast networks are controlled and enable selective signaling remains largely unknown (see Outstanding Questions). Through understanding the specificities and redundancies of redox signaling within the immune response in conjunction with other environmental stresses, it will be possible to uncover how responses to pathogen infection may be affected by a changing environment and how pathogens may take advantage of this. The development of new proteomic techniques has started to reveal a vast array of redox-sensitive proteins that hopefully will lead to new discoveries of how selective redox signaling networks orchestrate the plant immune response.

**Outstanding questions**

- How are redox signaling pathways rendered specific and reversible?
- Can newly emerging proteomic tools uncover the full extent of TRX redundancy and specificity in the plant immune response?
- To what extent do pathogens manipulate plant TRX systems?
- How do additional environmental stresses affect redox signaling networks of the plant immune response?

## Funding 

J.R.B. was supported by an EASTBIO Doctoral Training partnership (BB/M010996/1) from the Biological and Biotechnology Research Council and S.H.S by a grant from the European Research Council under the European Union’s Horizon 2020 research and innovation program (grant agreement No 678511).

## Notes

Steven H. Spoel: ORCID: 0000-0003-4340-7591; Twitter: @SpoelLab.

## Notes

J.R.B and S.H.S. wrote the paper.

The author responsible for distribution of materials integral to the findings presented in this article in accordance with the policy described in the Instruction for Authors (https://academic.oup.com/plphys/pages/general-instructions) is: Steven H. Spoel (steven.spoel@ed.ac.uk)
